# How would Australian women and people with a cervix like to access self-collection for cervical screening? Screening preferences from a national survey

**DOI:** 10.1007/s10552-026-02128-1

**Published:** 2026-02-10

**Authors:** Claire Bavor, Chloe Jennett, Emily Phillips, Louise Mitchell, Tessa Saunders, Lisa Whop, Angela Kelly-Hanku, Deborah Bateson, Julia M. L. Brotherton, Megan A. Smith, Claire Nightingale

**Affiliations:** 1 Evaluation and Implementation Science Unit, Centre for Health Policy, Melbourne School of Population and Global Health, University of Melbourne, Melbourne, Australia; 2 Cancer Elimination Collaboration, Sydney School of Public Health, Faculty of Medicine and Health, University of Sydney, Sydney, NSW Australia; 3 Yardhura Walani, National Centre for Aboriginal and Torres Strait Islander Wellbeing Research, Australian National University, Canberra, ACT Australia; 4 Kirby Institute, University of New South Wales, Sydney, NSW Australia; 5 Sydney Medical School, Faculty of Medicine and Health, University of Sydney, Sydney, NSW Australia

**Keywords:** Cervical screening, HPV, Self-collection, Models of screening, Self-sampling, Cervical cancer

## Abstract

**Purpose:**

In Australia, cervical screening is usually offered face-to-face through primary care. As self-collection offers flexibility in how and where screening can be accessed, we assessed participant preferences for flexible screening models.

**Methods:**

We recruited women and people with a cervix aged 24–74 years into a national survey (December 2023–April 2024) via a paid Meta campaign and community networks. Sociodemographic factors associated with a preference for appointment- or non-appointment-based models were assessed using logistic regression, stratified by age, < 50 and ≥ 50 years.

**Results:**

Among 9,586 respondents, the median age was 41 years, 67.9% lived in a major city, 82.5% were born in Australia, and 62.6% screened regularly. Most (82.6%) viewed flexible options for accessing screening as very important/important.

Respondents favored non-appointment-based compared to appointment-based models, with 53.5% of those < 50 (*n* = 4,842) and 49.5% of those ≥ 50 (*n* = 1,257) preferring to receive a swab in the mail when due. Non-appointment-based models were preferred by participants aged < 50 and ≥ 50 years who were never-screened (adjOR = 1.52, 95% CI = 1.18–1.96, *p* = 0.001; adjOR = 2.91, 95% CI = 1.67–5.09, *p* < 0.001), irregular screeners (adjOR = 1.58, 95% CI = 1.36–1.85, *p* < 0.001; adjOR = 1.52, 95% CI = 1.17–1.98, *p* = 0.002) and recently eligible for screening (adjOR = 1.64, 95% CI = 1.08–2.50, *p* = 0.02) compared to regular screeners. Convenience was the most common reason for participants’ preferred screening model (87.4% non-appointment-based; 55.1% appointment-based).

**Conclusion:**

Flexibility in how cervical screening can be accessed appeals to many screen-eligible people. Further research trialing different models assessing screening uptake and clinical pathways to follow-up care is needed.

**Supplementary Information:**

The online version contains supplementary material available at 10.1007/s10552-026-02128-1.

## Introduction

In Australia, women and people with a cervix aged 25–74 years are eligible for 5-yearly cervical screening using primary human papillomavirus (HPV) testing [1]. Since July 2022, the National Cervical Screening Program (NCSP) has recommended that individuals be offered a choice of HPV testing on a self-collected or a clinician-collected sample.[1] For self-collection, participants collect their own vaginal sample or are assisted to do this by a healthcare provider. HPV assays using polymerase chain reaction (PCR) have equivalent sensitivity for the detection of underlying high-grade cervical disease on self-collected and clinician-collected cervical samples (obtained via a speculum) [2].

Universal access to self-collection was introduced in the NCSP to address well-documented personal, social and structural barriers to screening participation.[3–7] Consistent with international data, self-collection is highly acceptable among Australian under- and never-screened women and people with a cervix as a more private, modest and convenient way of screening.[5, 8, 9] Uptake of self-collection within the NCSP reflects this, now accounting for more than 40% of all screening tests, with 50% of under- and never-screened participants choosing it [10].

In the NCSP, cervical screening, including self-collection, is primarily offered during a face-to-face consultation in primary care. It must be ordered by a healthcare provider with systems in place to ensure follow-up of results.[1] The practitioner-supported model is viewed as a strength of the NCSP as it provides an opportunity for healthcare providers to inform participants about screening options and support them through the screening pathway.[11] Follow-up rates among those requiring a clinician-collected sample for cytology following HPV (not 16/18) detection on a self-collected sample are high (85.1% within 6 months), as are colposcopy rates following HPV (16/18) detection among those using self-collection and clinician-collection (81.3% vs 80.5%, respectively, within 6 months).[12] However, socioeconomic, geographical, social, and personal barriers to accessing primary care, including rising out-of-pocket costs, [13, 14] also present barriers to screening using this model, resulting in inequities in screening participation and thus a need for flexible screening models [15, 16].

Self-collection offers flexibility that may address these barriers. Globally, self-collection has been offered in various clinical and non-clinical locations and facilitated by different types of healthcare workers, including non-medical providers.[17–19] Examples include community outreach with community healthcare workers [20] and Aboriginal community engagement workers;[8] mail-out;[21–23] opt-in models via online ordering [24] and pharmacy pick-up.[25] Other novel models include integrated breast, bowel and cervical screening [26] and telehealth.[27] Across studies, self-collection improves screening uptake among under- and never-screened population groups regardless of how it is offered; however, models with face-to-face invitation methods tend to be more effective than routine written invitations [17].

The National Strategy for the Elimination of Cervical Cancer in Australia calls for expanding who can offer screening and where and how it is offered to improve access.[16] The NCSP guidelines state that screening models, including those using self-collection, may be tailored to the needs of different communities.[1] However, it is unknown how Australian women and people with a cervix would like this flexibility to be used, including how they would like to obtain and return the self-collected swab. Therefore, the aim of this study was to assess preferences for participating in cervical screening and to explore the effects of demographic factors and screening history on these preferences.

## Methods

### Study design

This was a cross-sectional, online survey*.* It was conducted as a partnership between two national projects aiming to improve the equitable implementation of self-collection within the NCSP, *Supporting Choice for Cervical Screening* and *Screen Your Way* (an Indigenous-led team). The survey explored awareness, experiences of, and preferences for screening. This analysis reports data from questions exploring preferences. Other data are analyzed and reported separately, as are responses from Aboriginal and Torres Strait Islander respondents, in line with Indigenous data sovereignty principles [28, 29].

### Survey development

Sixteen items collected demographic and screening history variables and eight items assessed screening preferences (see Supplementary material 1). The latter were developed in reference to the strategic priorities outlined in Australia’s Elimination Strategy [16] and literature on screening models trialed globally.[8, 21, 24–27, 30] Most questions used multiple-choice and Likert-scale response options, and four included free-text fields. The whole survey took approximately 15 minutes. The survey used plain language and was reviewed for clarity by eight women in the Supporting Choice Community Advisory Panel.

### Study participants

Eligible participants included screen-eligible or soon-to-be eligible women and people with a cervix aged 24–74, living in Australia. Individuals who had had a total hysterectomy were excluded.

Qualtrics (Qualtrics, Provo, UT) settings prevented multiple responses from the same participant. Potentially fraudulent responses (i.e., bots and duplicate attempts) were removed using predetermined criteria, including survey duration of < 120 s or < 40% complete. Free-text responses were reviewed if more than five submissions were received within one minute or if the Qualtrics reCAPTCHA score was < 0.5 and Fraud score > 30 [31, 32].

### Data collection

The survey was open on Qualtrics between December 2023 and April 2024. Participants were recruited through a paid four-week Meta campaign (Facebook, Instagram) via Cancer Council Australia with adjustments made to the targeted audience over time to obtain a representative sample of the screening population. The survey was promoted through the project teams’ networks and by ACON, the largest community-based LGBTQ + health organization in New South Wales. Participants accessed the survey and plain language statement via an anonymous link and provided informed consent. Participants could enter a prize draw to win one of 24 $100 AUD gift vouchers.

### Data analysis

Quantitative data analysis was conducted in Stata (18.0, StataCorp LLC, College Station, TX). Respondents’ postcodes were mapped to remoteness and area-level socioeconomic status using the Accessibility/Remoteness Index of Australia Plus (ARIA +) 2021 and the Index of Relative Socioeconomic Disadvantage (IRSD) 2021.[33] Screening history was assessed using four variables (age, time since last cervical screening test, screening frequency, and reasons for being never-screened). Respondents were categorized as regular screeners, irregular screeners, never-screened (not screened, aged ≥ 27 years), and recently eligible for screening (not screened, aged < 27 years). Respondents were excluded if screening status could not be determined.

Data were summarized using descriptive statistics. The primary outcome was preference for a model of screening that did or did not involve an appointment in primary care (Table 1). Unadjusted binary logistic regression models were used to explore relationships between screening model preferences and demographic variables. Independent variables were then included in adjusted regression models if there was evidence of an association. Screening history was explored as a potential mediator by comparing models with and without screening history; however, evidence of a mediation effect was not observed (see Figure S5). The analysis was stratified by eligibility for breast and bowel screening (aged < 50 years vs ≥ 50 years).

**Table 1:** Definition of the binary variable used to compare preferences for non-appointment-based and appointment-based models of screening

**Non-appointment-based models** *Models of screening that do not require an appointment with primary care, including:*	**Appointment-based models** *Models of screening that do require an appointment with primary care, including:*
Clinic pick-up/drop-off	Telehealth with mail-out
Pharmacy pick-up	Telehealth with clinic pick-up
Online/phone order	Consultation with a healthcare worker
Mail-out when due	
Community events	
With bowel screening kit (for respondents aged ≥ 50 years only)	
At breast screening (for respondents aged ≥ 50 years only)	

This variable was derived by dichotomizing responses to the question “How would you most prefer to get the swab for self-collection?”

Qualitative data from free-text responses were analyzed using content analysis in NVivo (release 1.6.1, QSR International Pty Ltd).[34] Manifest, inductive coding was used to develop a coding framework by one author (CB). Codes were collapsed into higher-level themes reflecting the data. Codes and themes were revised after discussion with the research team (MS, CN) to develop the final framework. Responses could be coded more than once.

## Results

A total of 10,418 non-Indigenous individuals consented to the survey (responses from 555 Aboriginal and/or Torres Strait Islander individuals analyzed and reported separately). After removing 832 responses (405 ineligible; 412 not complete; and 15 potentially fraudulent), the final sample included 9,586 respondents (Table 2). The median age was 41 years, most had completed university (*n* = 5,866; 61.2%), lived in a major city (*n* = 6,122; 67.9%), and were born in Australia (*n* = 7,897; 82.5%). There were 5,999 (62.6%) regular screeners, 2,605 (27.2%) irregular screeners, 773 (8.1%) never-screened respondents, and 209 (2.2%) recently eligible respondents.

**Table 2:** Demographic variables by screening history

	Regular screeners^*^		Irregular screeners^†^		Never-screened		Recently eligible		Total	
	*n*	**%**	*n*	**%**	*n*	**%**	*n*	**%**	*n*	**%**
*Total*	5,999	62.6	2,605	27.2	773	8.1	209	2.2	9,586	100.0
*Age group*										
24–29 years	874	14.6	118	4.5	98	12.7	209	100.0	1,299	13.6
30–39 years	1,712	28.5	945	36.3	239	30.9	n/a	n/a	2,896	30.2
40–49 years	1,657	27.6	853	32.7	232	30.0	n/a	n/a	2,742	28.6
50–59 years	1,074	17.9	466	17.9	139	18.0	n/a	n/a	1,679	17.5
60–74 years	682	11.4	223	8.6	65	8.4	n/a	n/a	970	10.1
*Gender*^‡^										
Woman	5,936	98.9	2,571	98.7	766	99.1	199	95.2	9,472	98.8
Man	< 5	-	< 5	-	0	0.0	0	0.0	< 5	-
Non-binary/Different term	57	1.0	28	1.0	6	0.8	10	4.8	101	1.0
Prefer not to answer	5	0.1	< 5	-	< 5	-	0	0.0	8	0.1
*Sexuality*										
Straight	5,367	89.5	2,248	86.3	665	86.0	144	68.9	8,424	87.9
Bisexual	409	6.8	221	8.5	52	6.7	48	23.0	730	7.6
Gay/lesbian	89	1.5	64	2.5	26	3.4	9	4.3	188	2.0
Different term	85	1.4	46	1.8	21	2.7	< 5	-	156	1.6
Do not know	21	0.4	10	0.4	< 5	-	< 5	-	36	0.4
Prefer not to answer	28	0.5	16	0.6	6	0.8	< 5	-	52	0.5
*Intersex status*										
No	5,950	99.3	2,576	99.0	758	98.2	208	99.5	9,492	99.1
Yes	12	0.2	7	0.3	0	0.0	0	0.0	19	0.2
Do not know	29	0.5	19	0.7	14	1.8	< 5	-	63	0.7
Prefer not to answer	0	0.0	< 5	-	0	0.0	< 5	-	< 5	-
*Highest education completed*										
Primary or high school	718	12.0	318	12.2	132	17.1	29	13.9	1,197	12.5
TAFE, trade certificate or diploma	1,473	24.6	739	28.4	218	28.2	45	21.5	2,475	25.8
University	3,782	63.0	1,535	58.9	416	53.8	135	64.6	5,868	61.2
Other	5	0.1	< 5	-	< 5	-	0	0.0	7	0.1
Prefer not to answer	21	0.4	12	0.5	6	0.8	0	0.0	39	0.4
*State*										
ACT	199	3.3	88	3.4	30	3.9	7	3.3	324	3.4
NSW	1,847	30.8	849	32.6	229	29.6	66	31.6	2,991	31.2
NT	111	1.9	55	2.1	12	1.6	< 5	-	179	1.9
QLD	1,219	20.3	526	20.2	158	20.4	49	23.4	1,952	20.4
SA	357	6.0	132	5.1	52	6.7	11	5.3	552	5.8
Tas	215	3.6	88	3.4	24	3.1	5	2.4	332	3.5
Vic	1,334	22.2	552	21.2	179	23.2	56	26.8	2,121	22.1
WA	717	12.0	315	12.1	89	11.5	14	6.7	1,135	11.8
*Remoteness*										
Major city	3,887	69.0	1,596	64.8	510	69.8	129	68.6	6,122	67.9
Inner regional	1,190	21.1	581	23.6	148	20.2	40	21.3	1,959	21.7
Outer regional/ Remote/ Very remote	556	9.9	287	11.6	73	10.0	19	10.1	935	10.4
*Socioeconomic status quintile*										
1 Most disadvantaged	586	10.4	294	11.9	100	13.7	34	18.1	1,014	11.3
2	967	17.2	448	18.2	146	20.0	28	14.9	1,589	17.6
3	1,263	22.4	557	22.6	187	25.6	38	20.2	2,045	22.7
4	1,407	25.0	595	24.2	183	25.0	40	21.3	2,225	24.7
5 Least disadvantaged	1,406	25.0	568	23.1	115	15.7	48	25.5	2,137	23.7
*Country of birth*										
Australia	4,935	82.4	2,137	82.1	641	83.2	184	88.5	7,897	82.5
Other	1,052	17.6	467	17.9	129	16.8	24	11.5	1,672	17.5
*Number of years in Australia*										
Born Australia	4,935	82.6	2,137	82.3	641	83.2	184	88.9	7,897	82.7
Less than 9 years	174	2.9	92	3.5	21	2.7	9	4.3	296	3.1
10–20 years	315	5.3	120	4.6	47	6.1	10	4.8	492	5.2
More than 20 years	550	9.2	247	9.5	61	7.9	< 5	-	862	9.0
*Language at home*										
English	5,901	98.4	2,573	98.8	753	97.4	200	95.7	9,427	98.4
Other	93	1.6	31	1.2	20	2.6	9	4.3	153	1.6

Respondents whose screening status could not be determined were excluded from analysis. This includes those who responded ‘prefer not to answer’ for their last cervical screening test (*n* = 15) or were never-screened because they had never been sexually active (*n* = 155)

Column percentages are shown

^*^ Regular screeners include those who responded ‘I screen around about the time I am due’ to the question ‘How often do you attend cervical screening/have a Pap test?’

^†^ Irregular screeners include those who responded ‘I know that sometimes I leave it for too long between screens’ to the question ‘How often do you attend cervical screening/have a Pap test?’

^‡^ Response to the question ‘How do you describe your gender?’. Sex is not reported above as only participants whose sex recorded at birth was female were eligible for the survey

### Preferences for participating in cervical screening and self-collection

#### Perceived likelihood of screening because self-collection is now available

Among never-screened and recently eligible screeners, more than two thirds said they would be more likely to screen because self-collection is available (*n* = 529; 68.4%; *n* = 138; 66.0%, respectively; Table S1). Another 20% said its availability would not affect their decision to screen or not (*n* = 210; 21.4%) and a smaller proportion would still not screen (*n* = 41; 4.2%) or were not sure (*n* = 64; 6.5%).

#### Perceived importance of health service factors

Most respondents believed that having a choice over how they could do cervical screening (i.e., self-collection or clinician-collection) was very important/important (*n* = 8.052; 84.2%; Table S2) as was having flexible options for accessing screening (*n* = 7,847; 82.6%). Virtually all respondents thought it was very important/important (*n* = 9,030; 94.5%) to be able to talk to a healthcare provider about screening. Around 40% of respondents said they would prefer to discuss cervical screening with their usual doctor (*n* = 4,023; 42.0%) or a female doctor (*n* = 3,693; 38.5%). Another 11.6% said they would not mind who they spoke with (*n* = 1,109), and 5.9% said either a nurse, midwife or a health worker (*n* = 560; Table S3). It was very important/important for most respondents to have a healthcare provider who made them feel safe and comfortable (*n* = 9,480; 99.4%), to have a choice of a healthcare providers (*n* = 8,833; 92.7%) and to have a female healthcare provider (*n* = 7,283; 76.4%). Finally, it was very important/important to most respondents that a healthcare provider explained self-collection to them (*n* = 7,973; 83.5%).

#### Preferred location to self-collect a sample

Over half of the respondents would prefer to collect a sample at home (*n* = 5,039; 54.7%) and a quarter would prefer to do so at a clinic during an appointment (*n* = 2,331; 25.3%; Table S4). Notably, among respondents who were never-screened, collecting a sample at home was preferred by 70.8% (*n* = 523). Around 10% of all respondents said they would prefer collecting their sample at the place they picked up the swab (e.g., clinic, pathology collection center) (*n* = 943; 10.2%) or that they had no preference (*n* = 831; 9.0%).

#### Preferred way of returning the self-collection swab

Around 35% of respondents would prefer to return the self-collected swab by mail with a prepaid envelope (*n* = 3,456; 36.3%) or by dropping it off at a pathology collection center (*n* = 3,393; 35.7%; Table S5). Fewer preferred dropping it off at their healthcare provider’s clinic (*n* = 1,228; 12.9%). Returning the swab via prepaid mail was especially preferred among those who were never-screened (*n* = 334/762; 43.7%). Around 15% had no preference (*n* = 1,382; 14.5%).

### Impact of different models on perceived likelihood of screening on time

When considering whether or not they would be more likely to screen on time via different models (compared to the usual NCSP practitioner-supported model), most respondents said they would be more likely to screen on time if they received a self-collection swab in the mail when due (*n* = 7,297; 77.7%), could pick up a swab at the pharmacy (*n* = 4,889; 52.0%), or received a swab in the mail after a telehealth appointment (*n* = 4,757; 52.0%; Table S6). Respondents aged ≥ 50 years frequently said they would be more likely to screen on time if they received a self-collection swab with their bowel screening kit (58.1%; *n* = 1,496) or if they were offered it at a breast screening service (47.8%; *n* = 1,231).

### Most preferred model for accessing a self-collection swab for cervical screening

Respondents of all ages tended to prefer models that did not require an appointment (Tables 3 and 4). Around half of those aged < 50 (*n* = 3,585; 53.5%) and ≥ 50 (*n* = 1,247; 49.5%) said they would most prefer to receive a swab in the mail when due (Table S7). For those < 50, this was followed by picking up a self-collection swab at the pharmacy (*n* = 728; 10.9%) and ordering a swab online/over the phone (*n* = 690; 10.3%). For those aged ≥ 50, it was followed by receiving a swab with their bowel screening kit (*n* = 292; 11.5%) or picking up a swab from the pharmacy (*n* = 199; 7.8%).

**Table 3:** Factors associated with participants’ preferred model of obtaining a self-collection swab: participants aged less than 50 years old (*n* = 6,699)

	Most preferred screening model				Preference for non-appointment-based models			
	Appointment		Non- appointment		Unadjusted odds ratio (95% CI)	*p* value	Adjusted odds ratio (95% CI)	*p* value
	***n***	**%**	***n***	**%**				
*Total*	1,190	17.8	5,509	82.2				
*Age*								
24–29	245	19.5	1,011	80.5	Ref			
30–39	484	17.2	2,334	82.8	1.17 (0.99 – 1.39)	0.07	1.13 (0.94 – 1.36)	0.18
40–49	461	17.6	2,164	82.4	1.14 (0.96 – 1.35)	0.14	1.11 (0.92 – 1.34)	0.26
*Sexuality*								
Heterosexual	1,018	17.9	4,672	82.1	Ref			
Not heterosexual	164	17.5	774	82.5	1.03 (0.86 – 1.23)	0.76	–	-
*Highest education completed*								
School only	145	20.6	558	79.4	Ref			
TAFE, trade certificate or diploma	305	18.2	1,372	81.8	1.17 (0.94 – 1.46)	0.17	1.17 (0.94 – 1.46)	0.17
University	734	17.1	3,560	82.9	1.26 (1.03 – 1.54)	0.02	1.32 (1.08 – 1.61)	0.007
*Remoteness*								
Major city	779	17.8	3,597	82.2	Ref			
Inner regional	220	17.7	1,022	82.3	1.01 (0.85 – 1.19)	0.94	–	–
Outer regional, remote, very remote	117	17.3	561	82.7	1.04 (0.84 – 1.29)	0.73	–	–
*Socioeconomic status (quintiles)*								
1 Lowest SES	131	18.8	567	81.2	Ref			
2	192	17.3	920	82.7	1.11 (0.87 – 1.42)	0.42	–	–
3	264	17.9	1,213	82.1	1.06 (0.84 – 1.34)	0.61	–	–
4	282	18.0	1,282	82.0	1.05 (0.83 – 1.32)	0.68	–	–
5 Highest SES	246	17.1	1,195	82.9	1.12 (0.89 – 1.42)	0.33	–	–
*Country of birth*								
Australia	990	17.6	4,630	82.4	Ref			
Other	198	18.5	875	81.5	0.94 (0.80 – 1.12)	0.51	–	–
*Language*								
English	1,154	17.6	5,416	82.4	Ref			
Other	36	28.3	91	71.7	0.54 (0.36 – 0.80)	0.002	0.51 (0.35 – 0.76)	0.001
*Screening history*								
Regular screener	827	20.2	3,274	79.8	Ref			
Irregular screener	253	13.6	1,604	86.4	1.60 (1.37 – 1.87)	< 0.001	1.58 (1.36 – 1.85)	< 0.001
Never-screened	80	14.9	458	85.1	1.45 (1.13 – 1.86)	0.004	1.52 (1.18 – 1.96)	0.001
Recently eligible	30	14.8	173	85.2	1.46 (0.98 – 2.16)	0.06	1.64 (1.08 – 2.50)	0.02

Participants who stated in open-ended responses to the questions ‘Where would you most prefer to do self-collection (collect the sample yourself)’ and/or ‘If you were to do the test at home, how would you prefer to return the self-collection swab for it to be tested?’ that they would not participate in self-collection were excluded from this analysis (*n* = 49)

Row percentages are shown

The following independent variables were included in the adjusted regression model as there was strong evidence of an association in the unadjusted model: age, highest education completed, language and screening history

**Table 4:** Factors associated with participants’ preferred model of obtaining a self-collection swab: participants aged 50 years or older (includes at breast screening and with bowel screening kit) (*n* = 2,540)

	Most preferred screening model				Preference for non-appointment-based models			
	Appointment		Non- appointment		Unadjusted odds ratio (95% CI)	*p* value	Adjusted odds ratio (95% CI)	*p* value
	*n*	%	*n*	%				
*Total*	412	16.2	2,128	83.8				
*Age*								
50–59	217	13.4	1,400	86.6	Ref			
60–74	195	21.1	728	78.9	0.58 (0.47 – 0.72)	< 0.001	0.59 (0.48 – 0.74)	< 0.001
*Sexuality*								
Heterosexual	393	16.2	2,034	83.8	Ref			
Not heterosexual	18	18.4	80	81.6	0.86 (0.51 – 1.45)	0.57	–	–
*Highest education completed*								
School only	245	15.9	1,295	84.1	Ref			
TAFE, trade certificate or diploma	107	16.7	532	83.3	1.01 (0.73 – 1.39)	0.95	–	–
University	34	14.7	198	85.3	1.08 (0.81 – 1.44)	0.62	–	–
*Remoteness*								
Major city	245	15.9	1,295	84.1	Ref			
Inner regional	107	16.7	532	83.3	0.94 (0.73 – 1.21)	0.63	–	–
Outer regional, remote, very remote	34	14.7	198	85.3	1.10 (0.75 – 1.63)	0.63	–	–
*Socioeconomic status (quintiles)*								
1 Lowest SES	45	16.4	229	83.6	Ref			
2	67	15.8	358	84.2	1.05 (0.70 – 1.59)	0.82	–	–
3	81	16.3	417	83.7	1.01 (0.68 – 1.51)	0.96	–	–
4	88	15.1	493	84.9	1.10 (0.74 – 1.63)	0.63	–	–
5 Highest SES	105	16.6	526	83.4	0.98 (0.67 – 1.44)	0.94	–	–
*Country of birth*								
Australia	326	16.3	1,669	83.7	Ref			
Other	84	15.6	454	84.4	1.06 (0.81 – 1.37)	0.68	–	–
*Language*								
English	405	16.1	2,110	83.9	Ref			
Other	7	30.4	16	69.6	0.44 (0.18 – 1.07)	0.07	–	–
*Screening history*								
Regular screener	15	7.6	183	92.4	Ref			
Irregular screener	84	12.6	581	87.4	1.59 (1.22 – 2.06)	< 0.001	1.52 (1.17 – 1.98)	0.002
Never-screened	15	7.6	183	92.4	2.80 (1.63 – 4.81)	< 0.001	2.91 (1.67 – 5.09)	< 0.001

Note. Participants who stated in open-ended responses to the questions ‘Where would you most prefer to do self-collection (collect the sample yourself)’ and/or ‘If you were to do the test at home, how would you prefer to return the self-collection swab for it to be tested?’ that they would not participate in self-collection were excluded from this analysis (*n* = 20)

Row percentages are shown

The following independent variables were included in the adjusted regression model as there was strong evidence of an association in the unadjusted model: age and screening history. Language was not included in the adjusted regression model due to the small sample size

Screening models requiring an appointment were preferred by less than 20% of respondents in both age groups (Tables 3 and 4). The most popular appointment-based model among those aged < 50 years was receiving a swab in the mail after a telehealth appointment (*n* = 561; 8.4%; Table S8). For those aged ≥ 50 years, it was receiving a swab in the mail after a telehealth appointment or during an appointment (N = 157; 6.2% in each case; Table S8).

In adjusted regression analyses for respondents aged < 50 years, those who completed university were 1.32 (95% CI = 1.08–1.61, *p* = 0.007) times as likely to prefer non-appointment-based models compared to those who had not completed any post-school qualification (Table 3). Conversely, those who spoke a language other than English at home preferred appointment-based models compared to those who spoke English at home (adjOR = 0.51, 95% CI = 0.35–0.76, *p* = 0.001). Among respondents ≥ 50 years, those who were aged 60–74 years also preferred appointment-based models compared to those aged 50–59 years (adjOR = 0.59, 95% CI = 0.48–0.74, *p* < 0.001; Table 4).

In unadjusted and adjusted analyses, screening history was associated with a preference for non-appointment-based models compared to appointment-based models, regardless of age group. Among those aged < 50 years, those who were never-screened (adjOR = 1.52, 95% CI = 1.18–1.96, *p* = 0.001), irregular screeners (adjOR = 1.58, 95% CI = 1.36–1.85, *p* < 0.001), and recently eligible for screening (adjOR = 1.64, 95%CI = 1.08–2.50, *p* = 0.02) were more likely than regular screeners to prefer non-appointment-based models. Among those ≥ 50 years, those who were never-screened (adjOR = 2.91, 95%CI = 1.67–5.09, *p* < 0.001) and irregular screeners (adjOR = 1.52, 95%CI = 1.17–1.98, *p* = 0.002) were also more likely than regular screeners to prefer non-appointment-based models.

Sexuality, remoteness, socioeconomic status, and country of birth were not associated with a preference for non-appointment vs. appointment-based models of screening in the unadjusted or adjusted models in either age group.

### Reasons for most preferred model

The most common reasons for preferring non-appointment-based models were greater convenience (*n* = 6,746; 87.4%), availability outside work hours (*n* = 3,109; 40.3%), and being less expensive (*n* = 1,915; 24.8%). Being less embarrassing was selected by 23.1% (*n* = 1,781) of participants overall and more frequently among never-screened (*n* = 274, 42.2%), irregular (*n* = 655; 29.8%), and recently eligible screeners (*n* = 73, 42.0%) than regular screeners (*n* = 779, 16.6%). Similarly, a higher proportion of never-screened (*n* = 97; 14.9%), irregular (*n* = 204; 9.3%), and recently eligible respondents (*n* = 30; 17.2%) selected not having a trusted healthcare provider compared to regular screeners (*n* = 102; 2.2%; Figures S1, S2, S3, S4).

The most common reasons for selecting appointment-based models were more convenience (*n* = 890; 55.1%); preferring to talk to a healthcare provider (*n* = 565; 35.0%); and having a trusted healthcare provider (*n* = 454; 28.1%). Fewer never-screened respondents selected preferring to talk to a healthcare provider and having a trusted healthcare provider (*n* = 15; 15.5% in both cases) than regular (*n* = 438; 38.2; *n* = 350; 30.5%), irregular (*n* = 100; 29.5%; *n* = 82; 24.2%), and recently eligible respondents (*n* = 12; 40%; *n* = 7; 23.3%; Figures S1, S2, S3, S4).

### Content analysis of free-text ‘other’ responses

There were 806 free-text ‘other’ responses recorded. Responses to ‘other’ preferred healthcare providers to discuss screening with mostly cited a specific type of healthcare provider (*n* = 74/184; 40.2%), most frequently a gynecologist (*n* = 44/74; 59.5%; Table S9). Another 39.1% specified a female healthcare provider (*n* = 72/184). The most frequently specified ‘other’ location to collect a sample was a pharmacy (*n* = 14/76; 18.4%) and five (6.6%) said they would prefer to collect their sample at the clinic when using self-collection for the first time, but home thereafter (Table S10). To return the swab, the most common response was wanting multiple options available (*n* = 19/51; 37.3%; Table S11).

The most common ‘other’ reasons for preferring non-appointment-based models described wanting one less thing to do (267/560; 50.7%) or identified barriers to seeing a GP (*n* = 118/560; 22.4%; Table S12). ‘Other’ reasons for preferring appointment-based models included wanting to maintain a connection with a healthcare provider (*n* = 15/52; 25.9%) or being able to complete screening in one appointment/location (*n* = 13/52; 22.4%; Table S13). Thirty-five (6.0%) respondents said their experience with disability, chronic illness or being neurodiverse influenced their preference for an appointment-based (*n* = 4/52; 6.8%) or non-appointment-based (*n* = 31/560; 5.9%) model.

## Discussion

This is the first study to assess how Australian women and people with a cervix would like to participate in cervical screening using self-collection since its availability in the NCSP. Our findings highlight that respondents would value expanding how and where self-collection can be accessed, with over 80% reporting that having flexible options for accessing screening is important or very important to them.

Overwhelmingly, the most popular screening models were those not requiring an appointment, especially among respondents overdue for screening. While half of all respondents’ preferred to receive a swab in the mail when due for screening (mail-to-all), there was greater heterogeneity in the models favored by the remaining respondents; many not requiring an appointment (e.g., pharmacy pick-up, online/phone ordering, with bowel screening kit) were preferred to those requiring an appointment (e.g., telehealth in conjunction with either mail-out or clinic pick-up). These findings align with three international surveys of under- and never-screened women, where mail-to-all models were the most popular.[35–37] There is limited evidence of the effectiveness of mail-to-all models for cervical screening in Australia, as the only trial predates the availability of HPV screening and self-collection in the NCSP.[21] However, the uptake achieved among those overdue for screening was in line with international meta-analyses, showing mail-to-all, and opt-in (e.g., via mail, clinic pick-up) models improve participation compared to routine reminders for clinician-collection among under- and never-screened participants.[17, 21, 23] A mail-to-all model is used in the Australian National Bowel Cancer Screening Program; however, participation is only 41.7%.[38] An alternative access pathway enabling healthcare providers to provide bowel kits directly to eligible participants was introduced to boost uptake, based on findings that GP support is an important enabler.[39] Our finding that non-appointment-based models are preferred by those overdue for screening is consistent with findings from the Netherlands, where a higher proportion of women with no screening history used self-collection via mail-out than those previously screened (15% vs. 5.5%).[30] Several European countries with organized screening programs offer self-collection via mail-out either for under-screened (Finland, Denmark, France) or all screen-eligible participants (Netherlands, Albania, Sweden).[22] Reviewing these countries’ participation rates, when available, may assist in decisions about mail-out models as a supplement to usual care in the Australian context.

Our findings show the importance of considering the appropriateness of different screening models for different population groups, as we found that screening history, age, education, and primary language spoken at home were linked to respondents’ preferred screening model. Non-appointment-based models were more popular among respondents who were irregular screeners, never-screened, or recently eligible than regular screeners. Embarrassment and not having a trusted healthcare provider were more frequently endorsed as reasons for preferring non-appointment-based models among these respondents, consistent with these being well-documented barriers to screening.[5, 7, 9] Having completed university compared to having no post-school qualification was associated with preferring non-appointment-based models among respondents aged < 50 but not those aged ≥ 50. This may reflect several factors, including awareness of self-collection and health literacy.[40, 41] Convenience was the most common reason for respondents’ preferred model, regardless of screening history. The reasons that different models were seen as convenient varied; for example, non-appointment-based models were seen as more accessible, whereas appointment-based models were favored as the swab could be obtained and returned in the one location/time point. Another key motivator for preferring appointment-based models was wanting to talk to a healthcare professional. These models were relatively more popular among those aged 60–74 years, which may relate to them seeing GPs more frequently than younger age groups or having familiarity with face-to-face healthcare provision.[13] Appointment-based models were also preferred among participants aged < 50 years who spoke a language other than English at home. While several studies have identified community outreach and home-based screening models as ways of providing culturally safe screening,[42] this finding highlights the role of healthcare providers in supporting participation among this population group, regardless of how screening is offered.

The major study strength was the use of a targeted social media campaign to achieve a large sample size representative of the eligible screening population in terms of age, state, and remoteness.[43] However, recruitment via Meta may not have reached certain population groups, including those who are less likely to use social media, have low digital literacy, or are without Internet access. Furthermore, despite also using community networks to promote the study, participants born in another country (17.5% survey vs. 31.5% nationally) or who spoke a language other than English at home (1.9% survey vs. 22% nationally) were under-represented which may limit the generalisability of the findings.[44, 45] Similarly, a greater proportion of respondents in our study completed university (61.5%) compared to the proportion nationally (37.0% of women aged 15–74 years).[46] Other limitations include that screening history was self-reported so could be subject to recall as well as social desirability bias, potentially overestimating the number of regular screeners. Responses were also collected in regard to the primary screening test only and subsequent follow-up pathways may impact the acceptability of different models. Finally, implementing models of screening that address barriers to access experienced by Aboriginal and Torres Strait Islander people is critical for the equitable elimination of cervical cancer in Australia. We oversampled Aboriginal and Torres Strait Islander women and people with a cervix; their responses have been analyzed separately, according to Indigenous data sovereignty principles and so are excluded from this analysis.[28, 29]

### Policy and practice implications

While our findings indicate strong community support for non-appointment-based models, especially mail-out, it is important to consider the impact of these models on the current NCSP participation rate (74.6%) and follow-up, and thus whether they may sustain or improve program effectiveness.[15] Our findings can inform policy and practice, including trials to assess the uptake, acceptability, and appropriateness of different screening models. This will be important for understanding the impact of the intention-behavior gap on participation, a well-documented phenomenon across health behaviors.[47] It will also be important for determining when and how screening participants would like to access clinical support for screening models not delivered in-person, as even though 83% preferred a non-appointment-based model, over 90% of participants considered it important/very important to talk to a healthcare provider about screening. For many healthcare providers, discussing screening with participants is an important part of offering a safe and holistic service and ensuring that screening participants are referred to appropriate follow-up care for symptoms and abnormal screening results.[48] Implementation guidance will be needed to maintain screening quality in different settings though clear lines of clinical responsibility and robust pathways to follow-up care. Finally, the implementation of flexible models in the Australian NCSP, alongside usual care, will require sustainable funding solutions that enable medical and non-medical providers to offer screening in ways that best meet the needs of different population groups.

## Conclusion

Our findings show that greater flexibility in where and how self-collection could be accessed appeals to many Australian women and people with a cervix, but population groups differ in their preferences. Flexible screening models may address current barriers to accessing the NCSP and support the participation of those who are under- and never-screened. Our findings can be used to inform trials evaluating different models of screening to assess uptake and develop cervical screening pathways to ensure that cervical screening is effective, safe, and accessible.

## Supplementary Information

Below is the link to the electronic supplementary material.

Supplementary file1 (DOCX 45 kb)

Supplementary file2 (DOCX 207 kb)

## Data Availability

Datasets used and/or analysed during the current study are available from the corresponding author on reasonable request.
