# Supplementary material for: How would Australian women and people with a cervix like to access self-collection for cervical screening? Screening preferences from a national survey
Source: Cancer Causes Control. 2026 Feb 10;37(3):47. doi: 10.1007/s10552-026-02128-1 (PMC12891099; doi:10.1007/s10552-026-02128-1)

# Supplementary material

## Summary data tables and figures

Table S1. Never screened and recently eligible (aged <27 years) respondents perceived likelihood of taking part in cervical screening because self-collection is available (N=1,964)

|  | **Never screened** | | **Recently eligible** | | **Total** | |
| --- | --- | --- | --- | --- | --- | --- |
|  | **N** | **%** | **N** | **%** | **N** | **%** |
| More likely to screen | 529 | 68.4 | 138 | 66.0 | 667 | 67.9 |
| Does not affect decision to screen or not screen | 155 | 20.1 | 55 | 26.3 | 210 | 21.4 |
| Still would not screen | 36 | 4.7 | 5 | 2.4 | 41 | 4.2 |
| Not sure | 53 | 6.9 | 11 | 5.3 | 64 | 6.5 |

Table S2. Importance of health service factors (“How important are the following things to you when it comes to cervical screening?”)

|  | **Very important** | | **Important** | | **Not important** | | **Not sure** | |
| --- | --- | --- | --- | --- | --- | --- | --- | --- |
|  | **N** | **%** | **N** | **%** | **N** | **%** | **N** | **%** |
| Having a choice of how to do screening (N = 9,576) | 4,670 | 48.8 | 3,382 | 35.4 | 1,374 | 14.4 | 141 | 1.5 |
| Being able to talk to a healthcare provider about screening (N = 9,550) | 6,305 | 66.0 | 2,725 | 28.5 | 461 | 4.8 | 59 | 0.6 |
| Having clear and simple screening information (N = 9,551) | 7,465 | 78.2 | 1,987 | 20.8 | 85 | 0.9 | 14 | 0.1 |
| Having a healthcare provider explain self-collection (N= 9548) | 4,662 | 48.8 | 3,311 | 34.7 | 1,337 | 14.0 | 238 | 2.5 |
| Having a healthcare provider to help do self-collection if needed (N = 9,558) | 4,146 | 43.4 | 3,409 | 35.7 | 1,557 | 216.3 | 446 | 4.7 |
| Feeling safe and comfortable in clinic (N = 9,545) | 7,925 | 83.0 | 1,531 | 16.0 | 60 | 0.6 | 29 | 0.3 |
| Feeling safe and comfortable with the healthcare provider (N = 9,533) | 8,315 | 87.2 | 1,165 | 12.2 | 37 | 0.4 | 16 | 0.2 |
| Having a choice of healthcare provider for screening (N = 9,532) | 6,531 | 68.5 | 2,302 | 24.2 | 646 | 6.8 | 53 | 0.6 |
| Having a female healthcare provider for screening (N = 9,538) | 4,807 | 50.4 | 2,476 | 26.0 | 2,160 | 22.6 | 95 | 1.0 |
| Having flexible options for accessing screening (N = 9,497) | 4,293 | 45.2 | 3,554 | 37.4 | 1,423 | 15.0 | 227 | 2.4 |

Table S3. Preferred healthcare provider to discuss cervical screening with (N=9,580)

|  | **Regular screeners** | | **Irregular screeners** | | **Never- screened** | | **Recently eligible** | | **Total** | |
| --- | --- | --- | --- | --- | --- | --- | --- | --- | --- | --- |
|  | **N** | **%** | **N** | **%** | **N** | **%** | **N** | **%** | **N** | **%** |
| Usual doctor | 2,854 | 47.6 | 915 | 35.2 | 203 | 26.3 | 51 | 24.4 | 4,023 | 42.0 |
| Female doctor | 2,171 | 36.2 | 1,078 | 41.4 | 334 | 43.3 | 110 | 52.6 | 3,693 | 38.5 |
| I do not mind | 635 | 10.6 | 349 | 13.4 | 100 | 13.0 | 25 | 12.0 | 1,109 | 11.6 |
| Nurse | 158 | 2.6 | 160 | 6.1 | 81 | 10.5 | 15 | 7.2 | 414 | 4.3 |
| Health worker | 51 | 0.9 | 37 | 1.4 | 12 | 1.6 | 0 | 0.0 | 100 | 1.0 |
| Midwife | 20 | 0.3 | 13 | 0.5 | 10 | 1.3 | 3 | 1.4 | 46 | 0.5 |
| Other^*^ | 107 | 1.8 | 49 | 1.9 | 23 | 3.0 | 5 | 2.4 | 184 | 1.9 |
| Prefer not to answer | 0 | 0.0 | 2 | 0.1 | 9 | 1.2 | 0 | 0.0 | 11 | 0.1 |

* ‘Other’ free-text responses are described Table S11

Table S4. Preferred location to collect a sample (N=9,220)

|  | **Regular screener** | | **Irregular screener** | | | **Never- screened** | | | **Recently eligible** | | | **Total** | | |  |
| --- | --- | --- | --- | --- | --- | --- | --- | --- | --- | --- | --- | --- | --- | --- | --- |
|  | **N** | **%** | | **N** | **%** | | **N** | **%** | | **N** | **%** | | **N** | **%** | |
| At home | 2,824 | 48.8 | | 1,576 | 63.1 | | 523 | 70.8 | | 116 | 58.6 | | 5,039 | 54.7 | |
| During appointment | 1,713 | 29.6 | | 466 | 18.7 | | 106 | 14.3 | | 46 | 23.2 | | 2,331 | 25.3 | |
| Place picked up swab from | 603 | 10.4 | | 261 | 10.4 | | 57 | 7.7 | | 22 | 11.1 | | 943 | 10.2 | |
| No preference | 593 | 10.3 | | 177 | 7.1 | | 49 | 6.6 | | 12 | 6.1 | | 831 | 9.0 | |
| Other^*^ | 52 | 0.9 | | 18 | 0.7 | | 4 | 0.5 | | 2 | 1.0 | | 76 | 0.8 | |

* ‘Other’ free-text responses are described in Table S12

Table S5. Preferred healthcare method of returning swab (N=9,510)

|  | **Regular screeners** | | **Irregular screeners** | | **Never- screened** | | **Recently eligible** | | **Total** | |
| --- | --- | --- | --- | --- | --- | --- | --- | --- | --- | --- |
|  | **N** | **%** | **N** | **%** | **N** | **%** | **N** | **%** | **N** | **%** |
| Prepaid envelope | 2,072 | 34.8 | 982 | 38.0 | 334 | 43.7 | 68 | 32.7 | 3,456 | 36.3 |
| Pathology drop-off | 2,187 | 36.8 | 886 | 34.3 | 234 | 30.6 | 86 | 41.3 | 3,393 | 35.7 |
| Clinic drop-off | 794 | 13.3 | 325 | 12.6 | 79 | 10.3 | 30 | 14.4 | 1,228 | 12.9 |
| No preference | 862 | 14.5 | 382 | 14.8 | 115 | 15.0 | 23 | 11.1 | 1,382 | 14.5 |
| Other^*^ | 36 | 0.6 | 11 | 0.4 | 3 | 0.4 | 1 | 0.5 | 51 | 0.5 |

* ‘Other’ free-text responses are described in Table S13

Table S6. Likelihood of screening on time compared to now (practitioner-supported NCSP model)

|  | **More likely** | | **Same** | | **Less likely** | | **N/A** | |
| --- | --- | --- | --- | --- | --- | --- | --- | --- |
|  | **N** | **%** | **N** | **%** | **N** | **%** | **N** | **%** |
| Mail-out when due (N=9,394) | 7,297 | 77.7 | 1,523 | 16.2 | 363 | 3.9 | 211 | 2.2 |
| Pharmacy (N=9,398) | 4,889 | 52.0 | 3,051 | 32.5 | 1,182 | 12.6 | 275 | 2.9 |
| Mail-out after telehealth (N=9,449) | 4,757 | 50.3 | 3,821 | 40.4 | 572 | 6.1 | 299 | 3.2 |
| Clinic pick-up/drop-off (N=9,426) | 4,545 | 48.2 | 3,479 | 36.9 | 1,165 | 12.4 | 237 | 2.5 |
| Online/phone order (N=9,403) | 4,506 | 47.9 | 2,848 | 30.3 | 1,786 | 19.0 | 263 | 2.8 |
| During appointment (N=9,408) | 3,553 | 37.8 | 4,343 | 46.2 | 802 | 8.5 | 710 | 7.5 |
| Clinic pick-up after telehealth (N=9,432) | 2,457 | 26.0 | 4,720 | 50.0 | 1,968 | 20.9 | 287 | 3.0 |
| Community events (N=9,380) | 1,403 | 15.0 | 2,648 | 28.2 | 4,547 | 48.5 | 782 | 8.3 |
| With bowel screening kit (N=2,574) | 1,496 | 58.1 | 721 | 28.0 | 260 | 10.1 | 97 | 3.8 |
| At breast screening (N=2,577) | 1,231 | 47.8 | 920 | 35.7 | 315 | 12.2 | 111 | 4.3 |

Note. Responses for ‘with bowel screening kit’ and ‘at breast screening’ were from participants aged ≥50 years only.

Table S7. Most preferred model of screening among respondents aged less than 50 years (N=6,699)

|  | **Regular screeners** | | **Irregular screeners** | | **Never- screened** | | **Recently eligible** | | **Total** | |
| --- | --- | --- | --- | --- | --- | --- | --- | --- | --- | --- |
|  | **N** | **%** | **N** | **%** | **N** | **%** | **N** | **%** | **N** | **%** |
| **Non-appointment-based** | **3,274** | **79.8** | **1,604** | **86.4** | **458** | **85.1** | **173** | **85.2** | **5,509** | **82.2** |
| Mail-out when due | 2,147 | 52.4 | 1,038 | 55.9 | 279 | 51.9 | 121 | 59.6 | 3,585 | 53.5 |
| Pharmacy | 441 | 10.8 | 202 | 10.9 | 60 | 11.2 | 25 | 12.3 | 728 | 10.9 |
| Online/phone order | 355 | 8.7 | 227 | 12.2 | 93 | 17.3 | 15 | 7.4 | 690 | 10.3 |
| Clinic pick-up/drop-off | 329 | 8.0 | 137 | 7.4 | 26 | 4.8 | 12 | 5.9 | 504 | 7.5 |
| Community events | 2 | 0.0 | 0 | 0.0 | 0 | 0.0 | 0 | 0.0 | 2 | 0.03 |
| **Appointment-based** | **827** | **20.2** | **253** | **13.6** | **80** | **14.9** | **30** | **14.8** | **1,190** | **17.8** |
| Mail-out after telehealth | 384 | 9.4 | 112 | 6.0 | 53 | 9.9 | 12 | 5.9 | 561 | 8.4 |
| During appointment | 329 | 8.0 | 77 | 4.1 | 7 | 1.3 | 13 | 6.4 | 504 | 7.5 |
| Clinic pick-up after telehealth | 198 | 4.8 | 64 | 3.4 | 20 | 3.7 | 5 | 2.5 | 287 | 4.3 |

Note. Respondents who stated in free-text responses to the questions ‘Where would you most prefer to do self-collection (collect the sample yourself)’ and/or ‘If you were to do the test at home, how would you prefer to return the self-collection swab for it to be tested?’ that they would not participate in self-collection were excluded from this analysis (N=49)

Table S8. Most preferred model of screening among respondents aged 50 years or more (N=2,540)

|  | **Regular screeners** | | **Irregular screeners** | | **Never-screened** | | **Total** | |
| --- | --- | --- | --- | --- | --- | --- | --- | --- |
|  | **N** | **%** | **N** | **%** | **N** | **%** | **N** | **%** |
| **Non-appointment-based** | **183** | **92.4** | **581** | **87.4** | **183** | **92.4** | **2,128** | **83.8** |
| Mail-out when due | 821 | 49.0 | 341 | 51.3 | 95 | 48.0 | 1,257 | 49.5 |
| With bowel screening kit | 170 | 10.1 | 90 | 13.5 | 32 | 16.2 | 292 | 11.5 |
| Pharmacy | 126 | 7.5 | 57 | 8.6 | 16 | 8.1 | 199 | 7.8 |
| Clinic pick-up/drop-off | 117 | 7.0 | 40 | 6.0 | 14 | 7.1 | 171 | 6.7 |
| At breast screening | 80 | 4.8 | 24 | 3.6 | 13 | 6.6 | 117 | 4.6 |
| Online/phone order | 50 | 3.0 | 29 | 4.4 | 13 | 6.6 | 92 | 3.6 |
| Community events | 0 | 0.0 | 0 | 0.0 | 0 | 0.0 | 0 | 0.0 |
| **Appointment-based** | **15** | **7.6** | **84** | **12.6** | **15** | **7.6** | **412** | **16.3** |
| Mail-out after telehealth | 114 | 6.8 | 36 | 5.4 | 7 | 3.5 | 157 | 6.2 |
| During appointment | 127 | 7.6 | 25 | 3.8 | 5 | 2.5 | 157 | 6.2 |
| Clinic pick-up after telehealth | 72 | 4.3 | 23 | 3.5 | 3 | 1.5 | 98 | 3.9 |

Note. Respondents who stated in open-ended responses to the questions ‘Where would you most prefer to do self-collection (collect the sample yourself)’ and/or ‘If you were to do the test at home, how would you prefer to return the self-collection swab for it to be tested?’ that they would not participate in self-collection were excluded from this analysis (N=20)

*Figure S1. Reasons for preferred model of screening among regular screeners*

Note. Percentages do not sum to 100% as Respondents could select more than one option

*Figure 2. Reasons for preferred model of screening among irregular screeners*

Note. Percentages do not sum to 100% as Respondents could select more than one option

Figure S3. Reasons for preferred model of screening among never-screeners

Note. Percentages do not sum to 100% as Respondents could select more than one option

Figure S4. Reasons for preferred model of screening among recently eligible screeners

Note. Percentages do not sum to 100% as Respondents could select more than one option

## Content analysis of free-text ‘other’ responses

Table S9. ‘Other’ responses to preferred healthcare provider (HCP) to discuss screening with

| Category | N | Description | Example |
| --- | --- | --- | --- |
| Specific HCP | 74 | Gynaecologist, any HCP, nurse or midwife, not usual HCP, usual HCP, nurse practitioner, sexual health nurse | “I'll talk about it to anyone” (30-39 years, regular screener, P40) |
| Female HCP | 72 | Any female HCP, female doctor, usual female HCP | “I don’t mind as long as there *[sic]* female” (50-59 years, irregular screener, P59) |
| HCPs with specific qualities specified | 29 | HCPs with training/expertise, HCPs that are caring, kind, empathetic | “I would prefer to talk about cervical screening with a qualified healthcare professional (nurse, midwife or doctor) as long as I had a rapport with them, they understand how uncomfortable these tests can be and that I am safe, and that my consent is important and respected” (30-39 years, regular screener, P624) |
| HCP at a specific type of healthcare service | 16 | LGBTQI+ service, women’s health clinic, sexual and reproductive health service | “Nurse at family planning/ reproductive health clinic” (50-59 years, regular screener, P507) |
| No one | 13 | Do not want to speak to anyone | “No one” (30-39 years, never-screened, P639) |
| Would not do self-collection | 33 | Prefer to do clinician-collection, not eligible for self-collection, | “I would never want to participate in self-collection” (30-39 years, regular screener, P771) |

Table S10. ‘Other’ responses to preferred location to collect a sample

| Category | N | Description | Example |
| --- | --- | --- | --- |
| Home | 13 | Reason provided for preferring home, home because preference for obtaining swab would not be via GP | “At home with a telehealth appointment.” (30-39 years, regular screener, P84) |
| Pharmacy | 14 | Pharmacy | “Chemist” (40-49 years, regular screener, P411) |
| Clinic setting | 11 | Clinic if using self-collection for the first time then at home, reason for provided for clinic preference, women’s health clinics/sexual and reproductive health clinic | “At the clinic for the first time in case I have questions but from then on at home” (40-49 years, regular screener, P295) |
| Preference for multiple locations | 5 | Preference for more than one location, have options available | “At GP or home, dependant on mood at the time” (30-39 years, regular screener, P443) |
| A clean, private space | 3 | Clean, private, sterile, comfortable location | “Anywhere as long as the risk of the swab being contaminated is low and I can do the test somewhere properly and return it easily. It’s always hard when there are no proper bathrooms or rooms to do self-collection” (20-24 years, recently eligible, P363) |
| Would not do self-collection | 21 | Prefer to do clinician-collection, not eligible for self-collection | “I do not agree with self-testing” (24-29 years, regular screener, P682) |

Table S11. ‘Other’ responses to preferred method of returning swab

| Category | N | Description | Example |
| --- | --- | --- | --- |
| Preference for multiple ways | 19 | Preference for more than one way/location ‘or’, any of the above, have options available | “All of those options so I can choose the most convenient at the time.” (40-49 years, regular screener, P627) |
| Type of healthcare service specified | 3 | Women’s health clinic, hospital, urgent care clinic | “I live rurally and we don’t have easy access to a local doctor but we do have a hospital. So maybe at the local hospital would be easiest” (24-29 years, irregular screener, P313) |
| Chemist | 4 | Chemist | “Drop off to pharmacy” (40-49 years, irregular screener, P255) |

Table S12. Content analysis of ‘Other’ responses to reasons for preferred non-appointment-based model of screening (N=560)

| Category | N | Description | Example |
| --- | --- | --- | --- |
| Less to remember | 267 | Do not need to remember to do anything, receiving swab acts as a reminder, does not add to mental load, overcomes procrastination | “It prompts me rather than relies on me remembering” (24-29 years, irregular screener, mail-out when due, P127) |
| Barriers to seeing a GP | 118 | Hard to get GP appointment, live rurally, do not need to book an appointment, avoids interacting with a GP, cost of appointment, preferred HCP not available | “Trying to book a GP appointment is near impossible and I just don’t have the $$ right now. I don’t want to talk to anyone about it, just order it and send it off.” (30-39 years, never-screened, online/phone order, P660) |
| Convenience | 82 | More convenient, accessible location or hours, can be done at one location/at one time, avoids travel, part of routine, ensures swab arrives at right place | “More options -chemists are accessible” (30-39 years, regular screener, pharmacy, P102) |
| More empowering, private and safe | 39 | Anxiety about appointment or having cervical screening test, previous experiences of trauma, more comfortable/inclusive environment | “I have had traumatic experiences around pap smears and would rather reduce my exposure to medical professionals in this matter.” (20-29 years, irregular screener, online/phone order, P488) |
| Disability, chronic illness, neurodiversity | 31 | Addresses barriers to screening due to ADHD, PTSD, agoraphobia, autism, disability | “I have a disability that affects my memory and executive function, so I need to be able to test and return in one instance. Delays or having to take the swab somewhere, even to a post box, usually means I forget, panic or damage the sample.” (30-39 years, regular screener, clinic pick-up/drop-off, P367) |
| Confidence in ability to do self-collection | 7 | Confidence in ability to collect sample, feel well-informed | “I feel well enough informed to not have to discuss my preference with a healthcare provider” (30-39 years, regular screener, clinic pick-up/drop-off, P8) |
| Maintains swab quality | 7 | Ensures swab is sterile, does not get tampered with | “More confidence in the handling of the swab prior to collection and after drop off” (30-39 years, regular screener, clinic pick-up/drop-off, P261) |
| Maintains connection with HCP | 6 | Ask questions, continuity of care | “There is continuity of care if I pick up and drop off to the medical service I get my check-ups from. Avoid third party handling.” (24-29 years, regular screener, clinic pick-up/drop-off, P63) |
| Reduces waste/sustainable | 3 | Less waste, more sustainable | “I like that this is opt-in; if it was automatically sent out to anyone due for cervical testing I think that potentially generates a lot of single use plastic waste if it is sent out and not used.” (30-39 years, regular screener, online/phone order, P657) |

Table S13. Content analysis of ‘Other’ responses to reasons for preferred appointment-based model of screening(N=52)

| Category | N | Description | Example |
| --- | --- | --- | --- |
| Maintains connection with HCP | 15 | Provides ability to speak to HCP for guidance/support | “I feel like the knowledge of how to perform the self collection correctly is important and I trust my doctor. I would prefer he knows so that if I forget to bring it back he would remind me.” (24-29 years, irregular screener, during appointment, P387) |
| All done in one location/time | 13 | Do not need to remember to do anything, overcomes procrastination | “It means that it is done and dusted. Won't forget to post it or drop it off later.” (50-59 years, regular screener, during appointment, P94) |
| Convenience | 12 | More convenient, can be done at one location/at one time, part of a routine, avoids travel | “Do all the health things together” (30-39 years, irregular screener, during appointment, P504) |
| Disability, chronic illness, neurodiversity | 4 | Addresses barriers to screening due to ADHD, autism, disability | “I have ADHD so if I took it home I legit would never remember to return it. That’s why doing it at the clinic was so good because it didn’t require follow up from me” (30-39 years, regular screener, during appointment, P454) |
| Empowering, private or safe | 3 | Previous experiences of trauma, comfortable environment | “Have previously experienced pain, and want to be in an environment of comfort.” (24-29 years, regular screener, Mail-out after telehealth, P618) |
| Maintains swab quality | 2 | Ensures swab is sterile | “I want to ensure it’s sterile/clean and untampered with as opposed to getting it in the mail” (40-49 years, regular screener, Clinic pick-up after telehealth, P412) |
| Other | 3 | Barriers to seeing a GP, confidence in ability to do self-collection, reduces waste |  |

## Exploration of ‘screening history’ as a mediating variable

Figure S5. Directed acyclic graphs (DAGs) of the relationship between screening history and independent and dependent variables included in adjusted logistic regression models


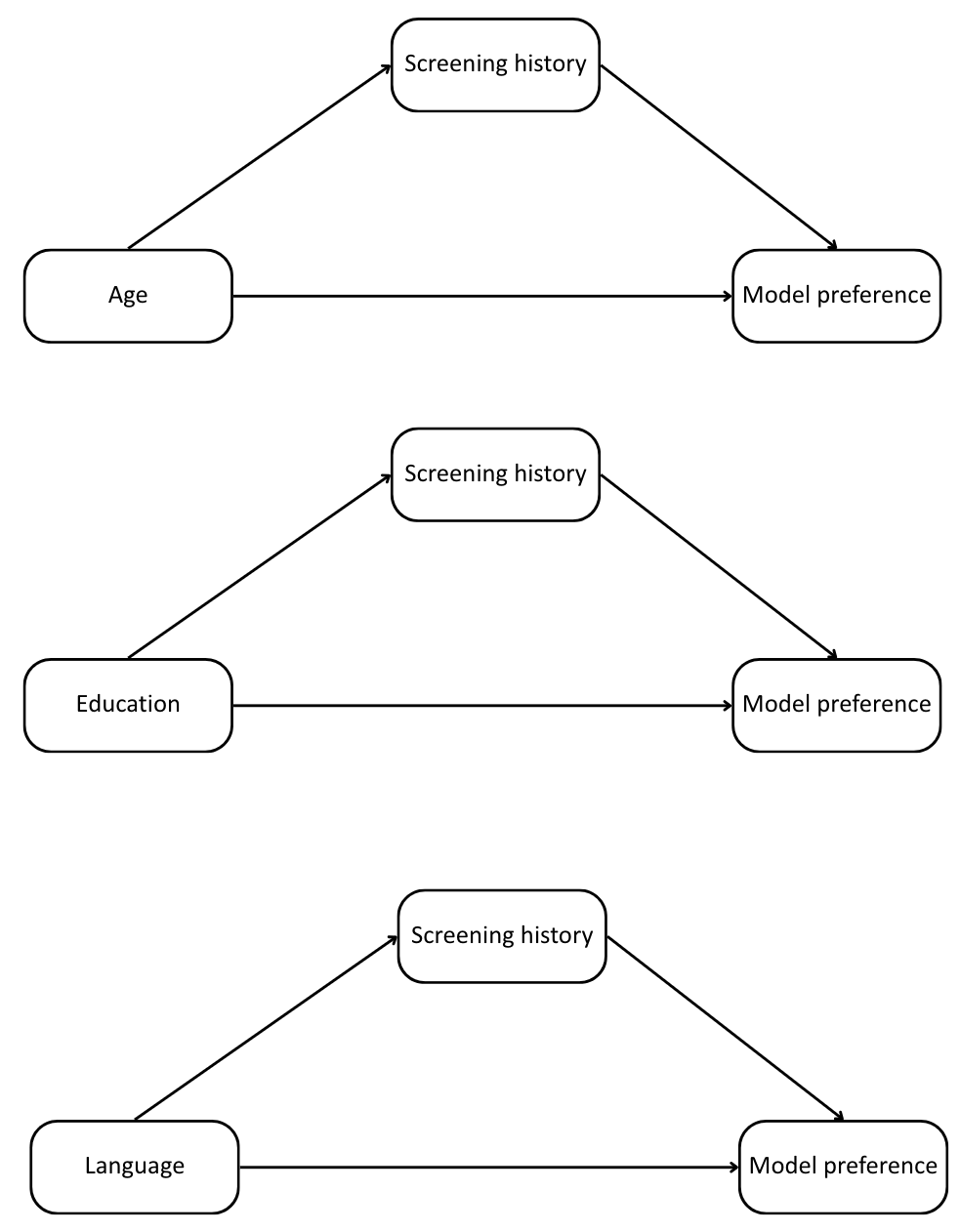

Supplement: Supplementary file 2 — Supplementary file2 (DOCX 207 kb) [file 10552_2026_2128_MOESM2_ESM.docx]
